# Vascular dysregulation in glaucoma: retinal vasoconstriction and normal neurovascular coupling in altitudinal visual field defects

**DOI:** 10.1007/s13167-023-00316-6

**Published:** 2023-02-16

**Authors:** Wanshu Zhou, Bernhard A. Sabel

**Affiliations:** Institute of Medical Psychology, Medical Faculty, Otto-Von-Guericke University of Magdeburg, Leipziger Straße 44, 39120 Magdeburg, Germany

**Keywords:** Glaucoma, Predictive Preventive Personalized Medicine (PPPM / 3PM), Retinal dysregulation, Neurovascular coupling, Ocular blood flow, Dynamic vessel analyzer, Predictive diagnostics, Targeted prevention, Vision restoration

## Abstract

**Purpose:**

Vision loss in glaucoma is not only associated with elevated intraocular pressure and neurodegeneration, but vascular dysregulation (VD) is a major factor. To optimize therapy, an improved understanding of concepts of predictive, preventive, and personalized medicine (3PM) is needed which is based on a more detailed understanding of VD pathology. Specifically, to learn if the root cause of glaucomatous vision loss is of neuronal (degeneration) or vascular origin, we now studied neurovascular coupling (NVC) and vessel morphology and their relationship to vision loss in glaucoma.

**Methods:**

In patients with primary open angle glaucoma (POAG) (*n* = 30) and healthy controls (*n* = 22), NVC was studied using dynamic vessel analyzer to quantify retinal vessel diameter before, during, and after flicker light stimulation to evaluate the dilation response following neuronal activation. Vessel features and dilation were then related to branch level and visual field impairment.

**Results:**

Retinal arterial and venous vessels had significantly smaller diameters in patients with POAG in comparison to controls. However, both arterial and venous dilation reached normal values during neuronal activation despite their smaller diameters. This was largely independent of visual field depth and varied among patients.

**Conclusions:**

Because dilation/constriction is normal, VD in POAG can be explained by chronic vasoconstriction which limits energy supply to retinal (and brain) neurons with subsequent hypo-metabolism (“silent” neurons) or neuronal cell death. We propose that the root cause of POAG is primarily of vascular and not neuronal origin. This understanding can help to better personalize POAG therapy of not only targeting eye pressure but also vasoconstriction to prevent low vision, slowing its progression and supporting recovery and restoration.

**Trial registration:**

ClinicalTrials.gov, # NCT04037384 on July 3, 2019.

## Introduction

Glaucoma, a leading cause of low vision [1, 2], is characterized by progressive visual field loss caused by degeneration of the visual pathway affecting the retina, optic nerve, and brain. For primary open angle glaucoma (POAG), risk factors include advanced age, non-white race, high myopia, family history of glaucoma, increased cup-to-disc ratio, cup-to-disc asymmetry, elevated intraocular pressure (IOP) [3, 4], and vascular dysregulation (VD) [5–7] especially in normal-tension glaucoma (NTG) [8]. Yet, diagnostics and treatment of glaucoma centers mainly on a single mechanism: IOP control. This neglects the systemic problem of blood flow regulation, and a pale optic nerve head, often interpreted as a sign of optic nerve degeneration, may be explained also by a reduction of ocular blood flow (OBF). Indeed, VD is the proposed mechanism of the “Flammer syndrome” which affects not only the eye and brain but also other organs with typical signs and symptoms [9, 10]. Women, slender people, people with indoor jobs, and academics suffer more likely from the Flammer syndrome [9], and psychological states (stress, anxiety) can trigger vasoconstriction and thus affect POAG progression and vision recovery [11–14].

Therefore, we should recognize that ocular vascular dysregulation and its treatment is of great value to better accomplish the goals of predictive, preventive, and personalized medicine (PPPM/3PM) in ophthalmology and rehabilitation of low vision.

Healthy vision depends not only on normal excitability of neurons in retina and brain, but it also depends on neurons’ metabolic homeostasis which requires a well regulated blood flow, both of which are tightly intertwined by “neurovascular coupling” (NVC) [15–17]. It is long known that in glaucomatous optic neuropathy (GON) [18, 19] and other eye [20, 21] and brain disorders [22–24], impaired blood flow is a key factor. Specifically in POAG, OBF is a major factor influencing progressive visual field loss with topographic (altitudinal) patterns of visual field defects [25]. Defect depth of the visual field is not uniform but varies considerably within the retina, ranging from areas of *absolute* defects (assumed “blind”), *relative* defects with reduced sensitivity, and regions with normal sensitivity to visual stimulation [26, 27]. Clinically, the spatial pattern of such “altitudinal” visual field loss also varies considerably between patients, and it can fluctuate over time [28, 29].

We may look upon vision loss, its topography, and its progression, as a combination of two problems: neuronal loss and vascular dysregulation. But the chicken-egg problem is this: which comes first? Although the relationship between localized visual field defects and corresponding retinal nerve fiber layer loss is well established [30–33], the cause-effect relationship of retinal blood flow and neuronal dysfunction is difficult to prove. Gasser et al. reported that blood-cell velocity in the nailfold capillaries of patients with NTG is reduced which supports the concept that vascular changes affect the whole body [25]. This is compatible with the idea that vascular dysfunction, not neuronal degeneration, may be the primary cause of glaucoma.

But what is possibly the cause of vascular dysregulation? Here, the observation of Flammer et al. [6, 9] are of interest who described that patients with the vascular dysregulation-associated “Flammer syndrome” tended to be ambitious, perfectionistic, and worrisome. This hints at the possibility that psychological factors such as stress, compulsiveness, and anxiety could be a major cause of vascular dysregulation, especially in glaucoma [11, 14, 34].

To explore this problem of vascular dysregulation from a different angle, we now studied glaucoma patients with a dynamic vessel analyzer (DVA) to characterize vessel dilation using fundus video recordings. It informs us of both vessel morphology and vessel dilation/constriction which—in turn—depend on neuronal activity, i.e., greater neuronal activity leads to greater dilation (NVC).

While previous DVA studies showed already that vessel dilation capacity is significantly altered in POAG and NTG [35–37], earlier results were inconsistent, possibly because only the large, primary branches of retinal vessels were measured without quantifying small vessels in higher-order peripheral branches. And there is no information how NVC relates to altitudinal visual field function.

By combining the analysis of vessel morphology (diameter, branch order), vascular dilation/constriction capacity, and visual field loss, we now explored in detail vessel morphology and NVC in the eye and their respective contribution to visual dysfunction. We reasoned that if the root cause of POAG is primarily of vascular origin, NVC should be intact but vessel morphology and/or function deficient. Because a detailed analysis of the eye’s vascular state is also a biomarker of the brain vascular state, and considering further that excessive mental stress-induced stress hormone release can reduce blood flow by vasoconstriction [11, 14, 38], a detailed vessel analysis helps also to link psychological states with eye and brain pathologies in glaucoma. It follows that detailed assessment of retinal vessel morphology and vessel’s dynamic response to neuronal activation is a novel approach to advance POAG diagnosis, help predict progression, and personalize glaucoma therapy.

## Methods

### Subjects

Thirty patients with POAG and 22 healthy controls were enrolled in a clinical trial of eye yoga from 8/2019 to 8/2021 (NCT04037384) as approved by the local Ethics Committee in accordance with the Declaration of Helsinki. Written informed consent was obtained from all subjects.

Patients and healthy control subjects (i.e., those with no known eye disease except for mild cataract) were recruited through public media using an online recruitment agency and screened for the following inclusion criteria for patients with POAG: (1) visual field defects due to POAG in at least one eye, (2) well-controlled IOP and blood pressure, and (3) refractive error between + 3.00 and − 6.00 diopter.

Exclusion criteria: (1) angle closure glaucoma, (2) severe cataract, (3) trauma or any other ocular disease (e.g., diabetic retinopathy, macular degeneration), (4) insufficient fixation ability or total blindness, (5) intraocular surgery within 6 months, (6) diabetes or fluctuating blood sugar, (7) blood pressure over 150/90 mmHg, or (8) addiction (alcohol abuse/smoking/drug dependency).

Inclusion criteria for healthy controls: no eye disease or eye surgery (mild cataract was acceptable).

Otherwise, the exclusion criteria for controls was the same as those of the POAG group.

### Visual field examinations

All subjects underwent visual field tests using the 30–2 pattern (6° stimulus distance) using stimulus size III/white (Twinfield-2 Perimetry; Oculus, Germany; equivalent to Goldman III size 0.43°).

### Dynamic vessel analysis

Using a dynamic vessel analyzer (DVA) (Imedos, Jena, Germany), retinal vessels were imaged [39–41] in a darkened room. After pupils were dilated with Mydriatics (1 ml solution of 5.0 mg of tropicamide, Pharma Stulln GmbH, Germany), the fundus was video recorded consisting of the following phases: (i) 50-s baseline measurement, (ii) three repeated 20-s stimulation periods of diffuse luminance 12.5 Hz flickers, and (iii) three 80-s post-flicker periods. Blood pressure (BP) was measured before and after DVA measurement, and IOP was measured prior and after pupil dilation and after completing DVA.

**Fig. 1:**
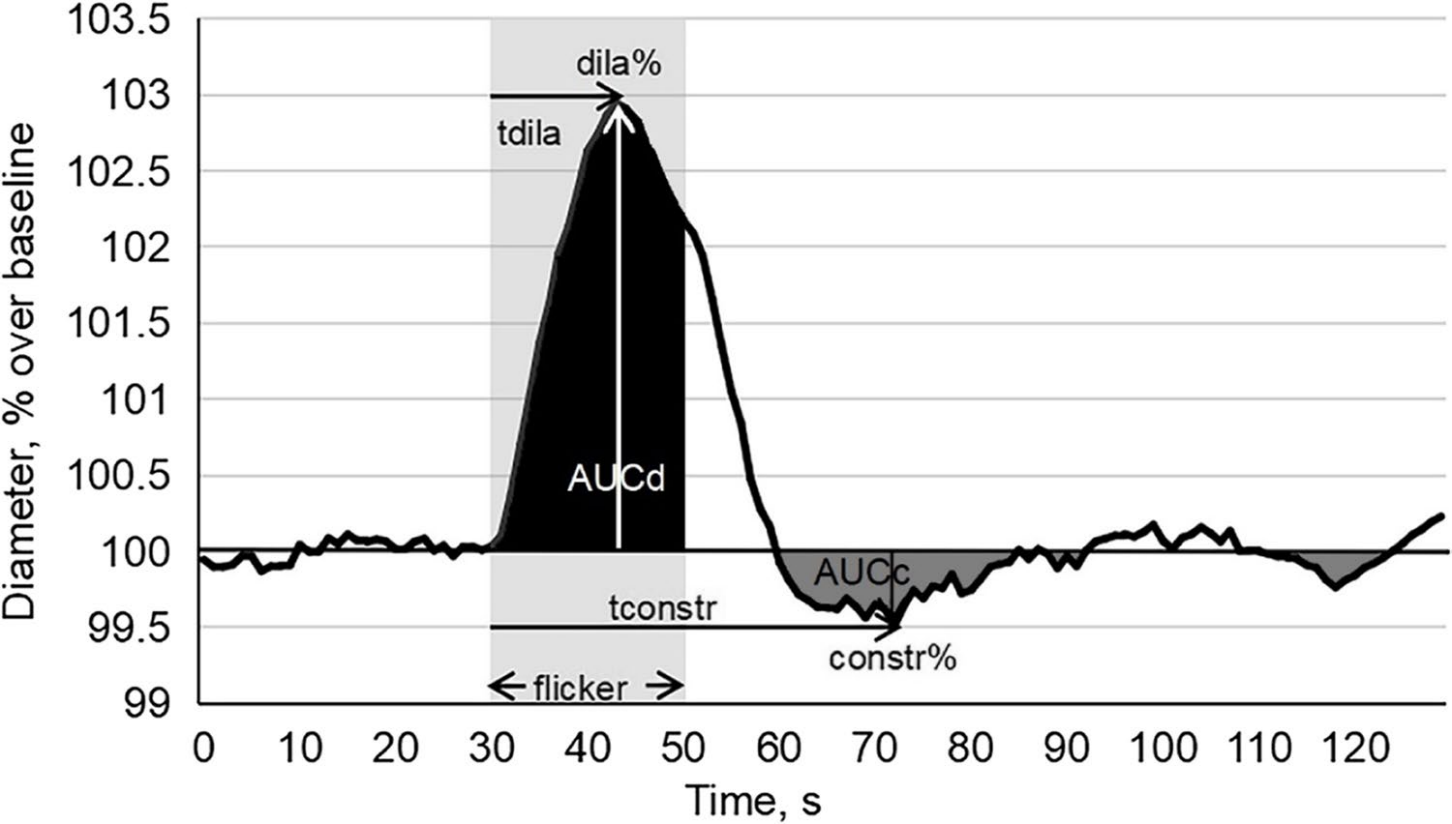
Parameters of retinal vascular response to flickering light stimulation: “maximal dilation” (dila%): peak dilatation during a 20-s flickering period compared to baseline; “time to maximal dilation”(tdila): time to peak dilatation after flickering onset; “maximal constriction” (constr%): peak constriction after flicker onset compared to baseline; “time to maximal constriction” (tconstr): time to reach peak constriction; “area under the dilation curve” (AUCd): area under the response curve above baseline during the flickering period; “area under the constriction curve” (AUCc): area under the response curve below baseline after flicker stimulation

Using DVA-software, vessel morphology and dilation dynamics were analyzed using absolute vessel diameter measurements (measuring unit: MU = micrometers in Gullstrand’s eye). To establish baseline diameter (100%), the three 30-s recordings before three flickers light presentation were pooled and averaged. Based on the subsequent diameter response to flickering light, expressed in percentage change over baseline, the following parameters were calculated: vessel diameter of arteries and veins and their maximal dilation (dila%); time to maximal dilation (tdila); maximal constriction (constr%); time to maximal constriction (tconstr); area under the dilation response curve above baseline during flickering (AUCd = dilation capacity); and area under the constriction response curve below baseline after flicker stimulation (AUCc = constriction capacity) (see Fig. 1 for details). Of note, we did not use a drug-holiday “wash-out” because this would be ethically problematic in cases with moderate glaucoma damage.

Unlike earlier studies, we measured vessel dynamics in multiple retinal locations and differentiated between branch hierarchies (larger vs. smaller vessels) and areas of visual field damage separately for intact eyes, if present, mild, moderate, and severe defects. Vessel segments were analyzed only if (i) located outside a circular area of one disc diameter from the optic disc center, (ii) more than one vessel diameter to neighboring vessels, (iii) if their image had sufficient contrast to surrounding tissue to allow artefact free recording, and (iv) no crossing, bend, or bifurcation in the measured segment.

### Topography of retinal vessel

**Fig. 2:**
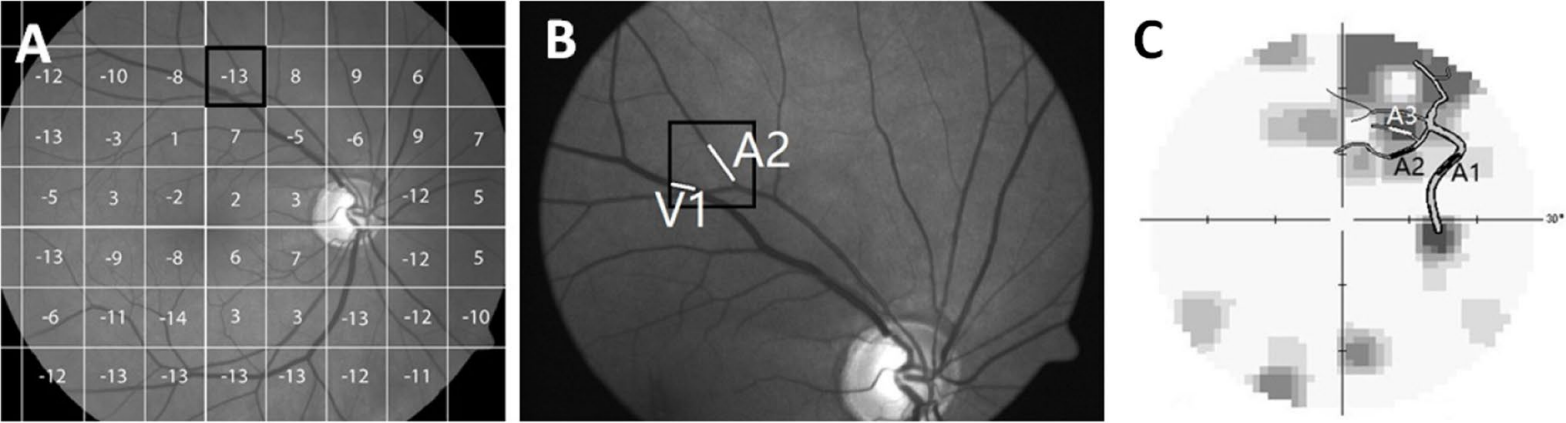
Compound analysis of retinal vessel tree, visual field function (in dB), determination of branch order for arteries (A1-3) and veins (V1-3). **A** Example of a retinal image with a superimposed 30–2 visual field test pattern (retina image resolution: 2452 × 2056 pixels; visual field size: 50° × 42°). The linear scaling was 2452**/**50 = 49 pixel/degree. The 30–2 pattern centers on the macula with the same linear scaling. The inter-point distances is 6° × 49 pixel/degree = 294 pixels. The retina image was divide into 6° × 6° squares centering on every visual field testing point. The pattern deviation result was assigned to the corresponding retina image squares (vertically flipped to match orientation). **B** Example how two vessels (A2, V1) located in the black square were assigned to their pattern deviation value (− 13 dB) according to the map shown in **A**. **C** Example of branch order analysis of levels 1–3 and how they were matched with the visual field defect values: A1 (first branch level) was located in the *mild defect* visual field area, A2 (second branch level) in a *moderate defect*, and A3 (third branch level) in a *severe* visual field sector

To match visual fields with retinal vessel images, retina photographs were first obtained by fundus camera (50°, Zeiss, Jena, Germany digitizing at 2452 × 2056 pixel resolution) to obtain linear scaling of 49 pixels/degree in an emmetropic eye. This image was superimposed on a 30–2 visual field plot of identical scaling (49 pixels/degree) [42] with Photoshop (Fig. 2A) and the center of the visual field was aligned to the fovea on the retina image [42]. This compound image was then divided into 6° × 6° squares with the visual stimulus located at the respective center. After vertical flip of the visual field chart, the retina fundus image and the specific pattern deviation value (visual field depth) could be matched (Fig. 2A). Vessel branch segment could thus be assigned a specific pattern deviation value according to their location and branch level (Fig. 2B). We then calculated the average branch segments per one of three pattern deviation sub-groups: mild defect > − 6 dB, − 6–12 dB moderate defect and severe defect ≤ − 12 dB. Eyes of subjects with POAG within normal limits or borderline were considered as “intact eye” (vessel segments of intact POAG eyes were not subdivided).

### Branch level analysis of retinal vessels

All vessel segments were assigned to one of three branch level sub-groups: branch order 1 is the vessel emerging from the papilla; branch order 2, the one first branching off from level 1 branch, and branch level 3 includes the next bifurcation and all higher branches (Fig. 2C). Of note, many level 3 branches were very small (often faint) and could not be measured, especially in the POAG group.

### Statistical analysis

Data were analyzed with SPSS 26 (IBM, New York, USA) and Matlab (MathWorks, Natick, USA), where each vessel was treated as an independent sampling point. The multiple-comparison problem was considered by defining significant differences of *p* < 0.005 (2-tailed) and *p* < 0.05 (2-tailed) as trend. Chi-square test was applied for gender analysis, Mann–Whitney *U*-test for two-group comparisons, and the Kruskal–Wallis test for multiple group comparison. Two-way ANOVA was used to assess interaction effects between two factors for average baseline diameter after removing extreme outliers (i.e., those at three-times of interquartile range from the lower or upper quartile in SPSS Box Plot).

### Demographic parameters, blood pressure, and IOP

The demographic parameters, blood pressure, and IOP are presented in Table 1. Mean age [standard error, SEM] was 65.2 [1.5] and 66.4 [1.6] for the control and POAG group, *p* = 0.599, and gender ratios (female/male) of both groups were comparable (control: 14/8; POAG: 16/14, *p* = 0.458) as was the blood pressure (control 132.1 [2.1]/82.6 [2.0] mmHg; POAG 135.0 [1.7]/82.9 [1.2] mmHg, n.s.). The average of three IOP-measurements was 12.3 [0.5] mmHg in patients with POAG, significantly lower than controls (14.3 [0.6] mmHg). The IOPs of subjects with POAG were well controlled by eye drops or surgery. The number of participants with well-controlled hypertension, hyperlipidemia, diabetes, and depression were also similar between groups (*p* > 0.9). The average IOP-lowering eye drops number was 1.6 [0.1] in the POAG group including those containing prostaglandins (*n* = 18), carbonic anhydrase inhibitors (*n* = 10), beta-adrenoceptor blockers (*n* = 10), and alpha 2 adrenergic receptor agonists (*n* = 6). Two subjects with hypertension in the control group were taking antihypertensives including angiotensin receptor blockers (*n* = 2), diuretic (*n* = 1), while 4 subjects with hypertension in POAG group were taking antihypertensive including angiotensin receptor blockers (*n* = 3), diuretics (*n* = 4), calcium channel blockers (*n* = 2), beta blockers (*n* = 1). The cup/disc ratio was 0.4 [0.02] and 0.7 [0.03] in the control group and POAG group, respectively. Mean deviation of the visual field was − 6.3 [0.9] dB in the POAG group.

**Table 1:** Demographic and medical parameters

	Control	POAG	p*
Number of subjects	22	30	
Number of eyes	43	57	
Age (SEM), year	66.4 [1.6]	65.2 [1.5]	0.599
Gender (female:male)	14:8	16:14	0.458
Systolic pressure (SEM), mmHg	132.1 [2.1]	135.0 [1.7]	0.351
DIASTOLIC pressure (SEM), mmHg	82.6 [2.0]	82.9 [1.2]	0.558
IOP (SEM), mmHg	14.3 [0.6]	12.3 [0.5]	0.021
Cup/disc ratio	0.4 [0.02]	0.7 [0.03]	< 0.001
Hypertension, *n*	2	4	0.973
Cardiovascular diseases, *n*	1	2	-
Diabetes, *n*	1	1	-
Hyperlipidemia, *n*	0	1	-
Depression, *n*	2	2	-
Mean deviation, dB		− 6.3 [0.9]	
IOP-lowering eye drop^†^, *n*		1.6 [0.1]	

^*^Chi-square test for gender analysis, Mann–Whitney *U*-test for other comparisons between control and glaucoma groups. ^†^One compound eye drop is considered as two types of eye drops

## Results

### Vascular morphology and dynamics

Vessel parameters are displayed in Table 2.

**Table 2:** Topographic features across different severity groups

	Control	POAG	Intact	Mild	Moderate	Severe	p*	*p*^†^	*p*^‡^
Artery									
Vessel number	383	479	99	271	65	45			
Diameter (SEM), MU	94.8 (0.9)	90.6 (0.7)	87.8 (1.6)	92.1 (0.9)	88.3 (1.5)	90.8 (1.9)	0.003	< 0.001	0.279
dila% (SEM), % over baseline	3.5 (0.1)	4.2 (0.1)	4.5 (0.3)	3.9 (0.2)	3.9 (0.4)	5 (0.6)	0.067	0.024^§^	0.118
constr% (SEM), % over baseline	− 2.6 (0.1)	− 3.5 (2.5)	− 3.7 (0.3)	− 3.5 (0.1)	− 3.2 (0.4)	− 3.8 (0.4)	< 0.001	< 0.001	0.066
AUCd (SEM), % * s	34.9 (3.1)	37.6 (1.7)	42.0 (3.1)	34.2 (2.1)	35.8 (4.6)	50.8 (7.2)	0.937	0.036^§^	0.250
AUCc, % * s	− 47.9 (3.0)	− 72.2 (3.6)	− 92.1 (10.3)	− 68.0 (4.3)	− 56.3 (9.5)	− 74.8 (9.1)	< 0.001	< 0.001	0.009^§^
tdila (SEM), s	13.4 (0.3)	12.4 (0.3)	12.5 (0.6)	12.3 (0.4)	12.3 (0.7)	12.6 (0.9)	0.012^§^	0.190	0.966
tconstr (SEM), s	50.3 (0.9)	49.8 (1.2)	45.1 (2.2)	49.2 (1.6)	59.3 (3.3)	51.2 (4.2)	0.69	0.084	0.039^§^
Vein									
Vessel number	371	513	98	311	51	52			
Diameter (SEM), MU	107.3 (1.5)	104.9 (1.1)	102.6 (3.2)	106.2 (1.3)	101.0 (3.4)	104.7 (3.6)	0.48	0.073	0.305
dila% (SEM), % over baseline	4.1 (0.1)	5.1 (0.2)	5.2 (0.4)	5.1 (0.2)	4.3 (0.5)	5.9 (0.8)	< 0.001	0.002	0.05^§^
constr% (SEM), % over baseline	− 2.1 (0.1)	− 2.7 (2.6)	− 2.8 (0.2)	− 2.5 (0.1)	− 2.8 (0.3)	− 3.6 (0.6)	< 0.001	0.004	0.141
AUCd (SEM), % * s	40.1 (1.5)	50.1 (2.0)	52.7 (4.6)	49.8 (2.2)	39.7 (5.4)	57.1 (9.7)	0.006^§^	0.007^§^	0.08
AUCc (SEM), % * s	− 34.9 (3.3)	− 47.4 (3.1)	− 59.9 (8.4)	− 42.3 (3.7)	− 46.0 (8.6)	− 55.7 (10.7)	< 0.001	< 0.00*1*	0.126
tdila (SEM), s	15.5 (0.2)	15.4 (0.2)	15.7 (0.5)	15.6 (0.3)	14.7 (0.6)	13.9 (0.7)	0.834	0.652	0.021^§^
tconstr (SEM), s	49.3 (1.9)	57.2 (1.5)	49.1 (3.3)	59.2 (1.9)	55.6 (5.0)	61.8 (4.8)	0.002	0.761	0.653

^*^Mann–Whitney *U* test between control and glaucoma groups, ^†^Mann–Whitney *U* test between control and intact groups, ^‡^Kruskal–Wallis comparison across mild, moderate, severe sub-groups*. dila%*, maximal dilation, peak dilatation during a 20-s flickering period compared to baseline; *tdila*, time to maximal dilation, time to peak dilatation after flicker onset; *constr*%, maximal constriction, peak constriction after flicker onset compared to baseline; *tconstr*, time to maximal constriction, time to reach peak constriction; *AUCd*, area under the curve above baseline during flicker stimulation; *AUCc*, area under the response curve below baseline after flicker onset; ^§^statistical trend

#### Vessel diameter

The average retinal artery diameter in patients with POAG was significantly smaller than controls (POAG 90.6 [0.7] MU; control 94.8 [0.9] MU). With 87.8 [1.6] MU, the intact eye of patients with POAG had the lowest diameter. While vein diameter was also somewhat reduced in POAG, this was not statistically significant when averaging all vessels. Thus, both arterial and venous (chronic) vasoconstriction was observed at baseline across the central retina in POAG, irrespective if there was vision or not in the different sectors.

#### Dilation/constriction capacity

Arterial dila% of POAG vessels tended to be slightly larger (percent over baseline: POAG 4.2 [0.1]%; controls 3.5 [0.1]%; *p* = 0.067) but significantly greater in veins (dila%: POAG 5.1 [0.2]%; control 4.1 [0.1]%, *p* < 0.001). These changes were only slightly dependent on the extent of vision loss (*p* = 0.05 in vein). Likewise, and logically so, absolute constriction was also larger in POAG (Table 2). While the total dilation capacity (AUCd) was unchanged in arteries, it was larger in veins (*p* = 0.006). The constriction capacity (the absolute AUCc) was significantly larger in POAG in both artery and vein, and the arterial AUCc was slightly dependent on the visual field depth.

Time to maximal dilation/constriction can be considered as an indicator of the systems stability which is expected to influence neuronal activity and synchronization because faster changes are more adaptive. We observed that glaucomatous arteries had normal dilation, but maximal constriction of the veins was significantly slower in POAG.

In sum, we uncovered in POAG smaller baseline diameters yet larger dilation and constriction during neuronal stimulation (flickering light). This suggests that despite smaller vessel diameters, dilation and constriction reach normal values showing that NVC is completely intact. Therefore, patients with POAG have vascular (vasoconstriction) problems but normal vessel dilation capacity following neuronal activation.

### Branch order effect on vessel response

#### Diameter

**Fig. 3:**
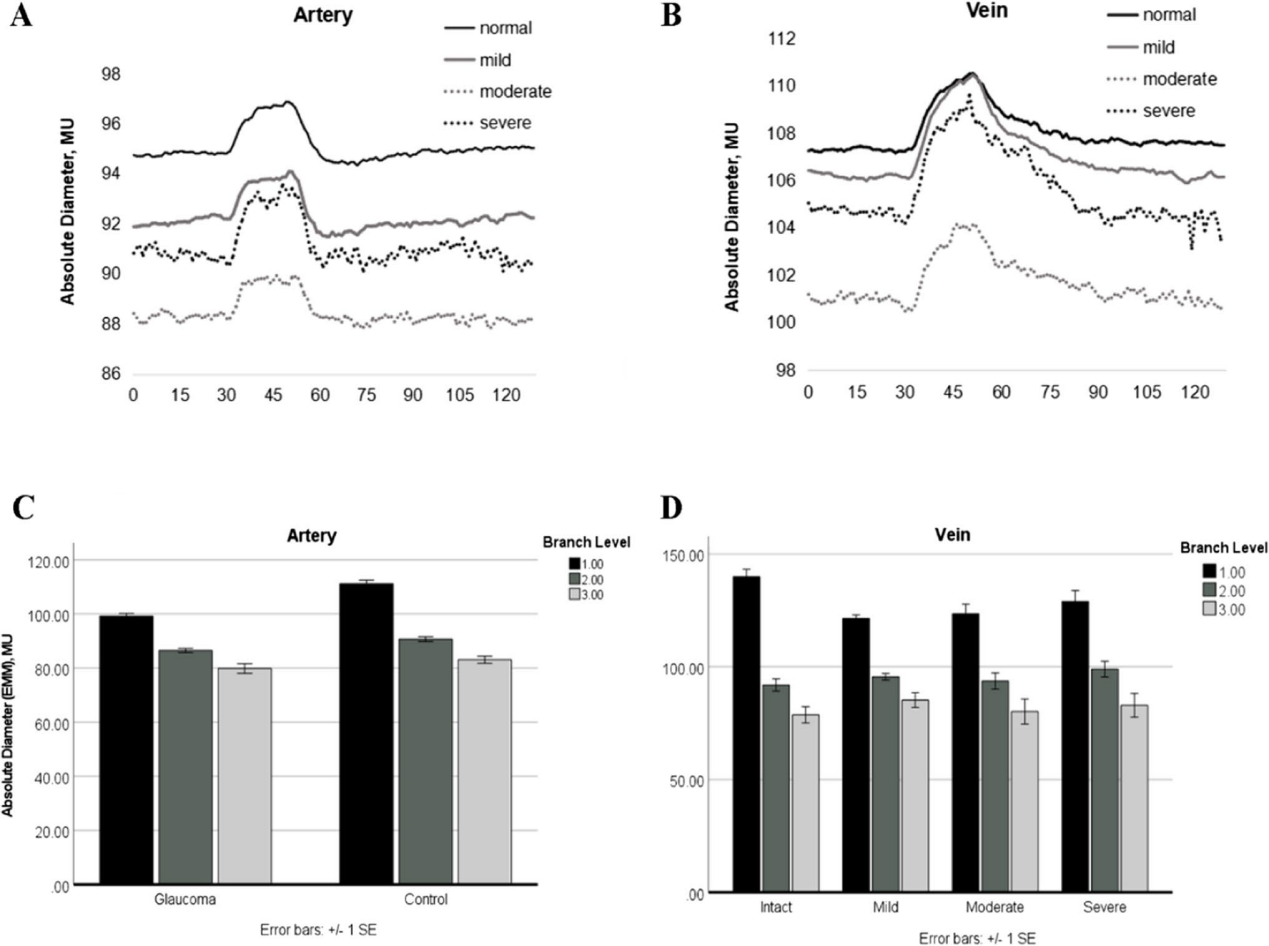
Upper panels: average diameter of artery (**A**) and vein (**B**) in response to flickering light provocation (dilation/constriction) displayed for the three glaucomatous visual field sub-groups compared to normal controls; surprisingly, vessels in the moderate visual field regions had the smallest diameter. Lower panels: **C** average artery baseline diameters for different branch order levels in the glaucoma and control group (*p* < 0.001); **D** venous diameter in glaucoma as a function of branch order and visual field defect depth (*p* = 0.004) (mean/S.E.M.)

The absolute diameters of higher order (smaller) branches were expectedly and significantly smaller in retinal arteries and veins in both groups (Table 3). The diameters of larger arteries (branch orders 1 and 2) were significantly smaller in POAG whereas for veins, only the largest vessel (branch order 1) was statistically smaller compared to controls. And there was an interaction effect of POAG vs. control and branch order with respect to arterial diameter (*p* < 0.001, Fig. 3C), visual field defect severities and branch order of venous diameter (*p* = 0.004, Fig. 3D).

**Table 3:** Vessel parameter for three branch level sub-groups

		1	2	3	*p*^‡^
Artery					
Vessel number	Control	93	204	87	
	POAG	159	273	48	
Mean deviation-(SEM), dB	POAG	− 3.7 (0.5)	− 4.4 (0.4)	− 3.1 (0.6)	0.117
Diameter-(SEM), MU	Control	112.6 (1.7)	91.7 (1.0)	83.7 (1.3)	< 0.001
	POAG	99.4 (1.0)	87.2 (0.8)	80.1 (1.5)	< 0.001
	*p*^†^	< 0.001	*< *0.001	0.128	
dila%-(SEM), % over baseline	Control	3.7 (0.3)	3.5 (0.1)	3.6 (0.3)	0.908
	POAG	4.2 (0.2)	4.1 (0.2)	4.4 (0.4)	0.31
	*p*^†^	0.236	0.617	0.060	
constr%-(SEM), % over baseline	Control	− 1.8 (0.1)23	− 2.8 (0.1)1	− 3.0 (0.2)1	< 0.001
	POAG	− 3.2 (0.2)	− 3.6 (0.2)	− 3.9 (0.4)	0.085
	*p*^†^	< 0.001	< 0.001	0.034^§^	
AUCd-(SEM), % * s	Control	39.8 (3.5)	35.1 (1.9)	29.5 (3.0)	0.051
	POAG	39.3 (2.8)	36.6 (2.3)	37.7 (4.5)	0.317
	*p*^†^	0.792	0.254	0.070	
AUC c-(SEM), % * s	Control	− 31.5 (2.9)23	− 52.9 (4.9)1	− 53.5 (5.9)1	0.004
	POAG	− 65.2 (6.4)	− 73.8 (4.5)	− 86.6 (14.4)	0.260
	*p*^†^	< 0.001	0.002	0.102	
tdila-(SEM), s	Control	43.9 (0.7)	43.1 (0.4)	43.3 (0.6)	0.241
	POAG	43.4 (0.5)	42.1 (0.4)	40.5 (0.9)	0.01^§^
	*p*^†^	0.407	0.070	0.009^§^	
tconstr-(SEM), s	Control	85.8 (2.8)	79.4 (1.7)	76.4 (2.8)	0.012^§^
	POAG	78.4 (2.0)	80.6 (1.6)	80.9 (3.7)	0.642
	*p*^†^	0.014^§^	0.650	0.196	
Vein					
Vessel number	Control	118	184	71	
	POAG	185	252	75	
Mean deviation-(SEM), dB	POAG	− 3.3 (0.4)	− 4.0 (0.4)	− 5.4 (0.8)	0.122
Diameter-(SEM), MU	Control	133.3 (2.2)	97.8 (1.6)	88.1 (2.5)	< 0.001
	POAG	124.9 (1.7) 23	96.8 (1.2) 13	82.6 (1.5) 23	< 0.001
	*p*^†^	0.001	0.851	0.207	
dila%-(SEM), % over baseline	Control	4.5 (0.2)3	4.1 (0.2)	3.7 (0.2)1	0.018^§^
	POAG	5.2 (0.2)	5.2 (0.2)	4.7 (0.5)	0.126
	*p*^†^	0.073	0.011^§^	0.102	
constr%-(SEM), % over baseline	Control	− 1.7 (0.1) 23	− 2.3 (0.1) 13	− 2.4 (0.3) 12	< 0.001
	POAG	− 2.1 (0.1)	− 3.0 (0.2)	− 3.1 (0.2)	< 0.001
	*p*^†^	0.003^§^	0.018^§^	0.014^§^	
AUCd-(SEM), % * s	Control	48.7 (2.5) 23	38.5 (2.2) 1	30.1 (2.6) 1	< 0.001
	POAG	54.2 (2.6)	49.4 (3.0)	42.2 (5.4)	0.003
	*p*^†^	0.388	0.055	0.119	
AUCc-(SEM), % * s	Control	− 24.4 (4.4) 23	− 37.7 (4.3) 1	− 45.2 (11.1) 1	< 0.001
	POAG	− 30.8 (3.1) 23	− 55.5 (5.0) 1	− 61.2 (9.8) 1	0.002
	*p*^†^	< 0.001	< 0.002	0.015^§^	
tdila-(SEM), s	Control	46.8 (0.4) 2	44.8 (0.4) 1	45.1 (0.6)	< 0.001
	POAG	46.3 (0.3)	44.8 (0.3)	44.9 (0.6)	0.02^§^
	*p*^†^	0.245	0.630	0.867	
tconstr-(SEM), s	Control	83.7 (3.3)	77.4 (2.7)	77.1 (4.2)	0.352
	POAG	87.6 (2.6)	88.4 (2.1)	82.0 (3.8)	0.34
	*p*^†^	0.388	0.002^§^	0.502	

^†^Mann–Whitney *U* test. ^‡^Kruskal–Wallis test. *dila%*, maximal dilation, peak dilatation during a 20-s flickering period compared to baseline; *tdila*, time to maximal dilation, time to peak dilatation after flicker onset; *constr%*, maximal constriction, peak constriction after flicker onset compared to baseline; *tconstr*, time to maximal constriction, time to reach peak constriction; *AUCd*, area under the curve above baseline during flicker stimulation; *AUCc*, area under the response curve below baseline after flicker onset. ^§^Statistical trend

#### Dilation/constriction capacity

Maximal dilation of arteries and veins were numerically, not statistically, slightly larger for POAG and were statistically comparable across branch order. AUCd was comparable across branch orders in artery, and vein AUCd decreased while the order increased. However, absolute constriction and AUCc were greater in first and second branch orders in POAG and increased while the branch order increased in control artery and in vein of both groups.

Time to maximal dilation/constriction: the smallest arterial vessels dilated faster in POAG, and vein of branch order 2 constriction was slower than control.

Of note: three extreme outliers were excluded from the analysis (one artery value in POAG group: 167.4 MU, one the in artery control group: 187.1 MU, and one value in artery branch level 3 group: 132.3 MU) when calculating the interaction effects of POAG vs. control and branch order with respect to arterial diameter processing. One extreme outlier was excluded in the vein branch order 2 group (198.7 MU) when calculating the interaction effects of POAG severity and branch order with respect to vein diameter because of limitations of the camera resolution of the DVA-camera. The DVA technique can only measure vessels > 55 MU, so the extreme outliers were those with large diameters in each group. The two-way ANOVA result was independent of the outlier removal (Fig. 3C). Of note, there is a large difference between the vessel samples in vein sub-groups (venous mild sub-group: 311, venous severe sub-group: 52), but the result of interaction effects of POAG severity and branch order with respect to vein diameter can at least show the trend because of the strong significance (*p* = 0.004, Fig. 3D).

## Discussion

A DVA analysis was employed to uncover the root cause of vision loss in POAG. The chicken-egg question is whether neuronal loss leads to OBF reduction or if vascular malfunction causes neuronal dysfunction. We reasoned that if vision loss is of neuropathological origin, then NVC (dilation) should fail when neuronal firing is triggered by flickering light. In contrast, if vision loss is of vascular origin, then dilation should be intact but vessel morphology (diameter) impaired according to the following scheme:

**Table:**
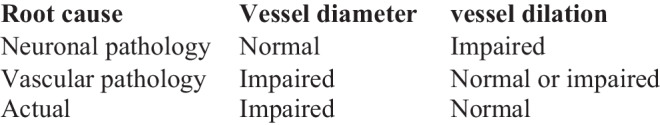

To solve this question, unlike previous studies of vessel dynamics, we studied subjects at later stages of POAG with DVA of many vessel segments, not just one or two main vessels [35–37, 43]. As we showed, arterial, but not venous, retinal vessel diameter was significantly smaller in POAG, yet patients had normal arterial dilation responses. We interpret this as a sign that the primary problem of POAG is not one of the vessels’ lack of dilation capacity (i.e., NVC) but rather one of reduced (resting state/baseline) vessel diameter.

While the absolute difference in vessel diameter between both groups seems small, the impact on OBF is big: according to Poiseuille’s law, vessel diameter has the 4th power influence on blood flow, where even a small (2–4 MU in our study) diameter loss can cause 100% − (100% − 4%)^4^ = 15% reduction of blood flow which is physiologically a lot. Our study thus confirms prior work showing blood flow reduction in glaucoma by reduced vessel caliber [44–46], vessel density loss [47–49], slower blood flow [50–52], and lower ocular perfusion pressure [53–55].

Our results contrast those of Garhöfer et al. [35] that flicker-induced vasodilatation in retinal veins is significantly diminished in POAG and those of Gugleta et al. [37] who reported a lower flicker light response in patients with POAG compared to patients with ocular hypertension. But our findings are consistent with Mroczkowska et al. [36] who reported maximal artery dilation to be larger in POAG (though not significantly so).

The source of the inconsistency could be various: differences in calculation method for dilation/constriction, different IOP levels, gender, IOP-lowering therapy, sampling method of diameters, retinal position of the samples, depth of vision loss at measured location, etc. However, unlike previous studies, we collected data from many vessels of different retinal locations in each eye, including all branch orders and diameters. This enabled us to study in detail the neurovascular response to visual (neuronal) activation in regions of different visual field depth and branch orders. As we showed, larger arterial and venous branches in POAG were significantly smaller which may be explained by the fact that larger vessels have more vascular smooth muscles.

While the association of the topography of the retinal nerve fiber layer (RNFL) loss and the location of visual field defects are known to be associated [30–32], our study is the first to topographically match the pattern of retinal vessel diameter and dilation ability (dysregulation) with local visual field dysfunction. Having quantified the vessel separately for three glaucoma severity areas (mild, moderate, severe visual field-defect areas), we found that their responses were rather similar with one possible exception: there is a hint that vessels in regions of *moderate* damage (“relative defects”) tend to have smaller venous response to flicker light than those in mild (expected) and in regions of absolute defect, which is counter-intuitive. While this finding is only a statistical trend without multiple-comparison adjustment (*p* = 0.05), this curious observation indicates that the different areas of damage may have different potentials to benefit from vascular therapies. Specifically, we expect visual field regions with partial (relative) defects to have more fluctuations and the greatest recovery potential as we have shown before [56]. If confirmed, intervention studies could focus their morphological (vessel status) and functional analyses (vision sensitivity) on those regions. In a speculative spirit, this could, in turn, be a way to better track progression of vision loss and estimating individual recovery potential more precisely, thus selecting and adjusting interventions more precisely on the basis of the individual patients visual field topography and vascular status, i.e., a more personalized approach to POAG therapy.

The fundamental question arises as to the possible *cause* of the (presumably permanent) retinal vessel constriction in POAG and the *effects* of it. Regarding the *cause*, we propose that in POAG smooth muscles tension is increased, reducing the diameter of the retinal vessels. Possible *reasons* for the vasoconstriction and reduced OBF can be different, but we believe that elevated stress hormone levels due to sudden or long-term mental stress gravely affect the retinal and brain microvasculature. Specifically, either long-term stress or sudden massive stress might lead to venous smooth muscle constriction, reducing venous vessel diameters with a sharply increasing retinal venous pressure [57].

Interestingly, Flammer, Konieczka et al. [6, 9, 10] discussed that patients with vascular dysregulation showing the “Flammer syndrome” tended to be ambitious, perfectionistic, and worrisome. As we have previously discussed, these personality features hint that psychological factors that increase stress, such as compulsiveness and anxiety, might be an underlying cause of vasoconstriction and vascular dysregulation, especially in POAG [11, 14, 34]. Therefore, the causal chain may be that massive, long-term (or sudden) mental stress leads to vasoconstriction by the smooth muscles, leading to lack of blood flow and insufficient energy delivery to visual system neurons. Furthermore, stress can trigger secondary complications including sleep disorders, depression, and (chronic low-grade) inflammation which, in turn, can have secondary effects of either silencing (surviving) neurons or triggering neurodegeneration in the retina and/or brain [26, 58].

Of course, these mechanisms are in addition to other known pathological alterations such as nitric oxide-cyclic guanosine monophosphate (NO-cGMP) signaling [59] and interpericyte tunneling nanotubes (IP-TNTs) [60] which are found in glaucomatous neurodegeneration in mice and in humans.

The causes of long-term stress can be many, including adverse childhood experiences, trauma exposure during childhood and adolescence, personal/financial stress, maladaptive levels of perfectionism, anxiety, low self-esteem, or compulsiveness. In fact, an increased prevalence of glaucoma was also reported in patients with mental disorders [61], and patients with POAG have an increased probability of more severe symptoms after mental-disorder manifestation [62].

Acknowledging this stress-related VD may offer new perspectives to develop glaucoma management from a 3PM perspective, i.e., combining traditional glaucoma therapies with relaxation techniques or other stress-reducing methods. In fact, this was already proposed by Strempel and her colleagues some 50 years ago [63, 64].

Specifically, and clinically most relevant, we propose that permanently reduced blood flow renders retinal (and brain) neurons hypo-metabolic with (partial) deprivation of oxygen, glucose, and other nutrients. However, when neural activation is induced with a strong kind of visual stimulation (e.g., flickering light during DVA), blood vessels throughout the retina can dilate and constrict normally. However, under normal visual conditions in everyday life, hypo-metabolic neurons remain silent, unable to fire action potentials for visual processing. Yet, their metabolism is sufficient for long-term survival with a potential to be reactivated/recover.

In summary, we propose that the (primary) root cause, or rate-limiting step, for visual functioning in POAG is of vascular origin (possibly because of excessive mental stress), which causes (secondary) long-term energy deprivation of neurons in retina and brain. This proposal is compatible with the “residual visual activation theory” [26] which proposed that silent neurons can—in principle—be reactivated because especially regions of moderate vision loss have some potential recovery/restoration as observed following vision training [65–68] or microcurrent neuromodulation [69–72]. In other words, and in a speculative spirit, moderately damaged regions may be those with the greatest diameter problem (at rest) which have the most “silent” neurons and the greatest recovery potential. It is an issue which requires further study.

Our study has several limitations. Firstly, our patients with POAG were on IOP-controlling topical medications which might affect our results. A “wash-out” period was not carried out because this would be ethically problematic in cases with moderate glaucoma damage (MD = − 6.3 dB). We found that the average retinal vessel diameter of our patients with IOP-lowering topical medications was significantly smaller than those of patients without eye drops (average diameter of anti-glaucoma topical medication sub-group: 89.8 MU, non-medication sub-group: 92.1 MU in artery, *p* = 0.029; diameter of anti-glaucoma medication: 103.6 MU, non-medication: 108.2 MU in vein, *p* = 0.016). The comparison is a rough estimation because other risk factors like POAG severity and anti-glacuoma surgery were not controlled. Besides, most of the patients were taking multiple IOP-lowering eye drops so further analysis is difficult. A well-designed study reported the artery diameter was not affected by the topical glaucoma medications compared to normal control [73]. The effects of IOP lowering medication and systemic medications on ocular blood flow is complex and controversial [74–82]. Future studies should try, as much as possible, to control the possible medication confound. Secondly, patients with hypertension were not excluded as long as their blood pressure was controlled by antihypertensives. Yet, we assume that this did not influence our results because the number of patients on anti-hypertensive drugs was comparable in both groups. Thirdly, while there may be an influence of POAG surgery on vascular health, in our sample, only three patients had such a history, where their trabeculectomy, phaco with i-stent implantation, or selective laser trabeculoplasty were performed in the anterior segment. Of note, however, future studies should keep this issue in mind, because ocular blood flow and retinal vessel response may change after eye surgery [83–86]. Finally, another limitation is that we may have underestimated the extent of small vessel diameter shrinkage: while many small vessels were visible by the observer, they were below DVA detection threshold due to insufficient (not recordable) blood flow, reducing the sample size when averaging. This could introduce a possible sampling bias. Yet, the very fact that this imaging problem exists confirms compromised blood flow in POAG.

## Conclusions

Using the DVA method, we were able to match retinal vessel responses to visual field defect depth in a group of patients with POAG. This revealed both arterial and venous (chronic) vasoconstrictions in POAG which limits blood flow at the resting state, continuously depriving neurons of retina (and brain) of oxygen and nutrients. We propose that depending on the extent of this hypo-metabolism, neurons may become either hypo-metabolic and inactive (“silent”), or they may die. However, despite vasoconstriction, retinal vessels in POAG are still able to dilate normally during neuronal activation (induced by visual stimulation with flickering light) which shows that their NVC remains intact. This observation is compatible with the hypothesis that vascular dysregulation, and not neuronal degeneration, is the root (primary) cause of vision loss in POAG.

On a conceptual level, our study contributes to a paradigm shift away from reactive medical service (IOP-driven decisions) toward a more personalized 3P-glaucoma treatment approach that considers a measurable vascular status in POAG. Regarding a predictive medicine, we would propose that psychological dispositions (long-term stress) can trigger a biological response (vasoconstriction) which then—if too long or too severe—leads to lowered neuronal energy states (in retina and brain), where mitochondrial stress may be part of the problem [38, 87]. This interpretation is compatible with different clinical phenomena, for example, fluctuations of visual field as a function of factors such as mental stress, time of day, atmospheric pressure, and high altitudes. And it could explain why visual training/rehabilitation, relaxation exercises, or microcurrent stimulation can improve visual field function again, possibly reactivating hypo-metabolic (silent) neurons [26, 27, 69]. Because stress was proposed to be an underlying cause—or at least a major risk factor—of POAG development and progression [11, 14], glaucoma should be viewed not only caused by eye pressure but also the consequence of complex interactions of mind (brain) and other body parameters (vascular system), with VD being the critical link between the two.

## Expert recommendations

In summary, we propose that the diagnosis of retinal vessel health, such as arterial and venous dilation and morphology, is a useful measure to help predict the development and progression of POAG and associated vision loss. A detailed vessel health diagnosis, in combination with psychological stress assessment, could serve to optimize therapeutic interventions for patients with POAG. This would be a more complex (holistic) and comprehensive, interdisciplinary, and personalized approach which considers psychological, biological, and pathological interactions. A better understanding of vessel health could possibly optimize existing therapies and allow for the development of novel interventions which not only focus on single parameter changes (IOP management) but follow a more comprehensive (multiple factors, such as mental stress, VD and IOP) “3P”-approach tailored to the individualized patient profile, considering both the psychological and physiological state of the patient’s individual profile. This more “holistic” understanding of POAG has important implications for all levels of primary (screening, early detection), secondary (treatment selection), and tertiary care (rehabilitation, lifestyle). It provides clinicians a “whole picture” understanding of POAG management, which might help especially patients with NTG and those where IOP lowering therapy fails to retain or restore vision.

## Data Availability

The data in the present study is available from the corresponding author on reasonable request.
